# How long did it last? You would better ask a human

**DOI:** 10.3389/fnbot.2014.00002

**Published:** 2014-01-27

**Authors:** Francesco Lacquaniti, Mauro Carrozzo, Andrea d’Avella, Barbara La Scaleia, Alessandro Moscatelli, Myrka Zago

**Affiliations:** ^1^Department of Systems Medicine, University of Rome Tor VergataRome, Italy; ^2^Centre of Space BioMedicine, University of Rome Tor VergataRome, Italy; ^3^Laboratory of Neuromotor Physiology, Santa Lucia FoundationRome, Italy; ^4^Cell Biology and Neurobiology Institute, National Research CouncilRome, Italy; ^5^Department of Cognitive Neuroscience, University of BielefeldBielefeld, Germany

**Keywords:** visual motion, biological motion, animate, inanimate, time perception, humanoid

## Abstract

In the future, human-like robots will live among people to provide company and help carrying out tasks in cooperation with humans. These interactions require that robots understand not only human actions, but also the way in which we perceive the world. Human perception heavily relies on the time dimension, especially when it comes to processing visual motion. Critically, human time perception for dynamic events is often inaccurate. Robots interacting with humans may want to see the world and tell time the way humans do: if so, they must incorporate human-like fallacy. Observers asked to judge the duration of brief scenes are prone to errors: perceived duration often does not match the physical duration of the event. Several kinds of temporal distortions have been described in the specialized literature. Here we review the topic with a special emphasis on our work dealing with time perception of animate actors versus inanimate actors. This work shows the existence of specialized time bases for different categories of targets. The time base used by the human brain to process visual motion appears to be calibrated against the specific predictions regarding the motion of human figures in case of animate motion, while it can be calibrated against the predictions of motion of passive objects in case of inanimate motion. Human perception of time appears to be strictly linked with the mechanisms used to control movements. Thus, neural time can be entrained by external cues in a similar manner for both perceptual judgments of elapsed time and in motor control tasks. One possible strategy could be to implement in humanoids a unique architecture for dealing with time, which would apply the same specialized mechanisms to both perception and action, similarly to humans. This shared implementation might render the humanoids more acceptable to humans, thus facilitating reciprocal interactions.

## INTRODUCTION

Robots are potentially very useful in several tasks where human resources may be limited or need to be spared, for example the assistance of elder people, care of children, physical therapy of disabled people, search and salvage of people in unsafe environments, or general help in daily life. These and similar tasks require a robot–human interaction, the interaction being proximal when the two (or more) partners are co-located (service robots placed in the same locale as humans; Mörtl et al., 2012), or remote when the partners are separated spatially and/or temporally (as in tele-operation; Goodrich and Schultz, 2007). In both cases, the interaction implies some sort of communication between the partners, and humanoid robots appear especially well suited to communication (e.g., Breazeal, 2003; Minato et al., 2004; Calinon et al., 2007). Humanoids are autonomous robots with anthropomorphic features, capable of mimicking human-like actions, and producing human-like reasoning (Goodrich and Schultz, 2007; Schaal, 2007).

Robot–human interactions present several formidable challenges, some of which are listed below. On the one hand, there is the hope that, in the future, humanoids will be as much human-like as possible, in order to be able to interact with people in the most natural manner (Jarrassé et al., 2012). For instance, it has recently been shown that the presentation of a humanoid face triggers an automatic orientation of spatial attention in humans, just as it does the presentation of a human face (Chaminade and Okka, 2013). On the other hand, paradoxically, the more human-like the appearance of a robot, the greater can be the social and emotional implications of its interaction with humans, because humans must accept the robot as an animate or quasi-living creature. As first hypothesized by Mori (1970), the sense of familiarity and general emotional response of a person who interacts with a robot may not increase monotonically with increasing anthropomorphism of the robot. At some point, the human reaction may suddenly become very negative when the robot closely but imperfectly reproduces a human being. Mori called this effect the “uncanny valley of eeriness.” The effect has recently been quantified by applying signal detection theory to the display of different types of computer-animated figures (Chaminade et al., 2007). By measuring the response bias of human observers toward “biological” or “artificial” categorization, it was found that the bias toward “biological” decreased with figures’ anthropomorphism, consistent with the “uncanny valley” hypothesis. Moreover, imaging the brain during the presentation of the different figures showed that the “biological” bias correlates positively with activity in regions involved in social cognitive processes such as mentalizing activity (e.g., the temporo-parietal junction; Chaminade et al., 2007). These findings therefore suggest that humans may not understand, feel empathy, and collaborate efficiently with humanoids which are highly anthropomorphic but are still perceived as artificial. In this respect, it may be more crucial that humanoids *behave* in a human-like manner, rather than they *resemble* humans. Thus, Nisky et al. (2012) proposed a Turing-like test for assessing alternative styles of handshake performed by a machine. The test is administered through a telerobotic system in which an interrogator holds a robotic stylus and interacts with another party, human or artificial. The inability of a human interrogator to distinguish between the handshake performed by a person and that performed by the machine indicates that the machine behaves in a human-like manner. There also exist standardized questionnaires to measure human perception of anthropomorphism, animacy, likeability, intelligence, and safety of robots (Bartneck et al., 2009).

Currently, much attention is being paid toward endowing robots with human-like movement features, under the premise that humans will collaborate better with robots which move like humans. Indeed, some progress is been made in implementing human-like movements in some robots (Schaal, 2007; Sugimoto et al., 2012). Although the movements of most current robots are still a caricature of human movements attesting the difficulty of imitating us, a promising approach appears the application of the movement primitives extracted from human subjects (for instance, by means of principal component analysis) to transfer the features of human movement to a robot (Choe et al., 2007; Moro et al., 2012). Even more challenging appears the task of endowing robots with the ability to understand the manner in which humans perceive the world, another critical prerequisite for cooperative interactions between robots and humans. In humans, action is strictly coupled to perception. Because of substantial sensori-motor delays, most motor responses of humans cannot be simply reactive to a given external event, but must be somehow predictive, that is, the responses must incorporate knowledge about the forthcoming evolution of the event (Zago et al., 2009). In fact, it is known that perception and action share, at least in part, common representations and common knowledge (Prinz, 1997; Rizzolatti and Craighero, 2004; Choe et al., 2007). To accomplish shared tasks, robots and humans should interact knowing what each other is doing. Of course, robots could be endowed with their own, idiosyncratic knowledge-based perceptual system, but presumably they would interact more successfully with humans if they shared with humans a similar knowledge-based perceptual system, as well as temporal cognition (Maniadakis and Trahanias, 2011).

As remarked above, neural processing of sensory information is fraught with substantial delays (considerably longer than those typically present in robots), but the brain somehow compensates for them, so that we are unaware of constantly living in the past, so to speak (Nijhawan, 2008). Thus, neural responses lag behind the adequate visual stimulus by 50–100 ms in several visual cortical areas, including the primary visual cortex (Schmolesky et al., 1998). The flash-lag effect is a visual illusion in which a flashed object appears to lag behind a moving object, when physically the two objects are co-localized at the instant of the flash (Nijhawan, 1994). One explanation of the effect is that the visual system is predictive, accounting for neural delays by extrapolating the trajectory of the moving stimulus into the future (Nijhawan, 1994). Alternatively, however, visual awareness might be postdictive, so that the percept attributed to the time of an event is a function of what happened during the last 80 ms after the event (Eagleman and Sejnowski, 2000).

Moreover, processing delays can differ significantly among different sensory channels: for instance, acoustic stimuli are processed much faster than visual stimuli. Nevertheless, when we see and hear someone snapping his or her fingers, we perceive the event as unitary. The sight and sound appear simultaneous, as if the brain synchronized internally the corresponding visual and auditory signals.

Human perception is a vastly complex performance, but the temporal dimension is essentially ubiquitous because perceived actions and events unfold in time. Animals, people (and less frequently, inanimate objects) are seldom static, and our sensory landscape is typically dynamic, populated by moving targets. The critical point to be considered for implementing human-like perceptual abilities in robots is that human perception of elapsed time for actions and events is two-sided, being both quite precise and quite inaccurate. In general, the precision (variable error) exhibited by humans in processing time information across an extremely large range of temporal intervals is striking. The Weber ratio is about 10% over 10 orders of magnitude of the base time interval, from the microsecond timing of sound localization to the 24-h period of events evolving with a circadian rhythm (Gibbon, 1977; Buhusi and Meck, 2005). On the other hand, the accuracy (constant error) of estimates of the duration of events can be surprisingly poor, perceived duration often being very loosely related to the physical duration of the event. Subjective durations can be systematically overestimated (time dilation), or underestimated (time compression), and the performance is highly context-dependent (Fraisse, 1963; Mauk and Buonomano, 2004; Eagleman et al., 2005; Eagleman, 2008).

Here we briefly consider some examples of time distortions in human perception, and dwell more extensively on the special case of the effects of visual motion on subjective duration. Also, we will mainly discuss the perception of events unfolding over scales of tens to hundreds of milliseconds, because these time scales are common to typical motor actions. We will argue that human perception of time is strictly linked with the way humans control their own movements. Therefore, implementation of human-like perception in humanoids will also depend on the progress being made in implementing human-like motor control.

## DISTORTIONS OF PERCEIVED TIME

Perceived duration is affected by several factors, as shown by the behavior in response to the presentation of simple visual stimuli. When humans are asked to judge the duration of a flash, they often make systematic errors. Thus, a simple reduction in the visibility of a flash leads to underestimating its duration (Terao et al., 2008). In addition to luminance, also the numerosity and size of the stimuli affect time estimates: stimuli with larger magnitudes in these non-temporal dimensions are judged to be temporally longer (Xuan et al., 2007).

Also the extent to which the stimulus can be predicted affects time perception (Ulrich et al., 2006). If a given stimulus is flashed repeatedly, the duration of the first stimulus appears longer than that of the successive stimuli (Pariyadath and Eagleman, 2007). By the same token, a stimulus which stands out as different from all the others in a series appears to last longer than the other stimuli, even though they all have the same physical duration (Tse et al., 2004). Another well-known factor affecting perceived duration is represented by the amount of attention paid to the stimulus: the higher the level of attention, the longer is the perceived duration (Tse et al., 2004; New and Scholl, 2009).

Another kind of distortion in time perception occurs when the stimulus is presented close in time to the execution of a movement performed by the observer. For instance, a visual stimulus flashed just after an eye saccadic movement appears to last longer than normal (Yarrow et al., 2001). On the other hand, duration judgments are compressed during eye saccades (Morrone et al., 2005). In the latter case, observers largely underestimate the time interval elapsed between two brief visual stimuli which are flashed near in time to a saccade. Another example of distortion is represented by the apparent compression of the time epoch which has elapsed between the execution of a simple movement (such as a button press) and a subsequent event (such as a beep or flash; Haggard et al., 2002). Subjective duration of intervals filled with task-irrelevant events is longer than that of empty intervals, the increase depending on the complexity of the perceptual processing required by the event (Buffardi, 1971).

In addition to those listed above, several other factors affect time perception, such as arousal and emotional levels (Hancock and Weaver, 2005), stimulus complexity (Roelofs and Zeeman, 1951; Schiffman and Bobko, 1974), concurrent task complexity (Macar, 1996), and temporal uncertainty (Zakay, 1992). Some of these distortions can be accounted for within the “counter/accumulator” model of time perception (Creelman, 1962; Fraisse, 1963; Treisman, 1963; Gibbon, 1977; Brown, 1995). In the context of this conceptual model, internal pulses are generated, collected, and integrated during the presentation of a stimulus. The output of the counting process is then compared with memorized time representations to estimate the overall duration of a given time epoch. In this framework, an increment of the variable (e.g., size, luminance, novelty, or arousal) which is critical for time perception in a given task would lead to a transient increase in the rate of the internal clock. Consequently, the accumulator would sum a larger number of pulses in a given time epoch, and the stimulus duration would be judged accordingly longer.

Also other models have been proposed to account for time distortions. In one such model, subjective duration parallels the amount of neural energy (or the total amount of neural activity) used to encode a stimulus (Pariyadath and Eagleman, 2007). In higher cortical areas, neuronal firing rate tends to decrease in response to repeated presentations of the stimuli, and this may explain why subjective duration is longer for the first than the subsequent stimuli in a row. Still another model posits that timing is a distributed process, being encoded by the spatio-temporal patterns of activity in multiple neural populations (Mauk and Buonomano, 2004). A stimulus typically engages hundreds of excitatory and inhibitory neurons, and also triggers time-dependent processes (e.g., synaptic plasticity). As a consequence, the state of the neural network is different when another stimulus arrives slightly later. The difference in the network activity produced by the second and first stimulus may code for the time interval separating the two stimuli.

## PERCEIVED DURATION OF VISUAL MOTION

Considerable progress has been made in the phenomenological knowledge in this field of research over the last few years (see Eagleman, 2008; Zago et al., 2011a). Not only is visual motion common in daily life, but it is also so salient that the changes over time of the visual stimuli may index the passage of time by themselves: how much time has passed can be determined by counting these indices (Brown, 1995). This is closely related to the “counter/accumulator” model mentioned above. As one would expect from the application of this model, visual motion is typically associated with misperceptions of elapsed time. Thus, it is known that the perceived duration of a moving stimulus is longer than that of a stationary stimulus having the same physical duration (Lhamon and Goldstone, 1975; Brown, 1995; Kanai et al., 2006), and the apparent duration of the moving stimulus increases with increasing speed (Leiser et al., 1991; Brown, 1995; Beckmann and Young, 2009; Kaneko and Murakami, 2009). Indeed, according to the “counter/accumulator” model, faster stimuli would generate a greater number of events, and the longer would be the corresponding estimated duration. Also the specific kinematic profile of the moving target can affect temporal judgments. For instance, a constant-speed motion seems to last longer than a decelerating motion, which in turn seems to last longer than an accelerating motion (Matthews, 2011).

The specific dynamic factor associated with visual motion which is responsible for the time distortion is still unclear. According to one hypothesis, stimulus speed would be directly involved: the apparent duration would increase proportionally with the logarithm of speed (Kaneko and Murakami, 2009). According to an alternative hypothesis, however, temporal frequency rather than speed would be the critical factor, as shown by the fact that time dilation can be induced simply by flickering a stimulus, with no need for motion (Kanai et al., 2006).

In addition to the visual effects induced in real-time by a moving stimulus, there are also after-effects. For example, the prolonged exposure to a pattern moving at constant speed affects the perceived speed of subsequent moving patterns: the perceived speed of that stimulus and all slower speeds are reduced, while the perceived speed of faster stimuli is increased (Thompson, 1981; Smith and Edgar, 1994; Hammett et al., 2005; Hietanen et al., 2008). These after-effects can be accounted for by current models of speed processing. Perceived speed is thought to be based on the ratio of the outputs of low-pass and band-pass temporal filters, corresponding to a low- and high-speed channel whose sensitivities decay exponentially over time (Smith and Edgar, 1994; Hammett et al., 2005). Adaptation to a fast speed produces a change in filters sensitivities resulting in a drop of the ratio, and perceived speed is slower. Instead, following adaptation to a slow speed, the change in filters sensitivities results in an increase of the ratio, and perceived speed is faster. Similar mechanisms are presumably at play in time perception. Thus, the apparent duration of a dynamic stimulus is reduced in a region of visual space following motion adaptation (Johnston et al., 2006), and the effect of this adaptation can be spatially selective either in retinal (Bruno et al., 2010) or external coordinates (Burr et al., 2011).

## PERCEPTION IS TUNED TO DOMINANT PROPERTIES OF THE ENVIRONMENT

Perceptual biases are not simply the result of idiosyncratic neural processing of sensory signals, but often reflect *a priori* hypothesis made by the brain about the functional significance of the signals. In particular, it is thought that, under evolutionary and developmental pressure, the brain adapts to be tuned to the statistical properties of the signals to which it is exposed most frequently (Simoncelli and Olshausen, 2001). For instance, the statistical distribution of target speeds in the natural environment is skewed toward low values. A prior preference for slow speeds can result in severe misperceptions, as when the speed of a visual target is underestimated with small target size or low contrast. These misperceptions are accounted for by the fact that the noisier the signal (as with small, low-contrast targets), the greater is the influence of the prior assumption of low speed (Weiss et al., 2002).

Prior hypotheses about the environment can be revealed by the presence of illusions and misperceptions under unusual conditions, but their functional utility lies in the ability to improve the performance under ecological conditions. One such prior hypothesis concerns the ubiquitous and highly predictable effects of Earth’s gravity (Zago et al., 2009). Gravity plays a major role in determining the orientation of objects in the environment, and therefore the structure of our visual field. Most natural images are anisotropic, with more image structure at orientations parallel or orthogonal to the direction of gravity in a fronto-parallel plane (Hansen and Essock, 2004). These image anisotropies are often matched by corresponding anisotropies in perceptual responses, consistent with the hypothesis that the brain takes into account the statistics of the environment. The well-known “oblique effect” refers to the fact that contours are better discriminated when they are oriented vertically or horizontally (cardinal directions) than when they are oriented obliquely (Appelle, 1972). Similarly, motion direction is better discriminated along cardinal than oblique axes (Ball and Sekuler, 1987).

**FIGURE 1:**
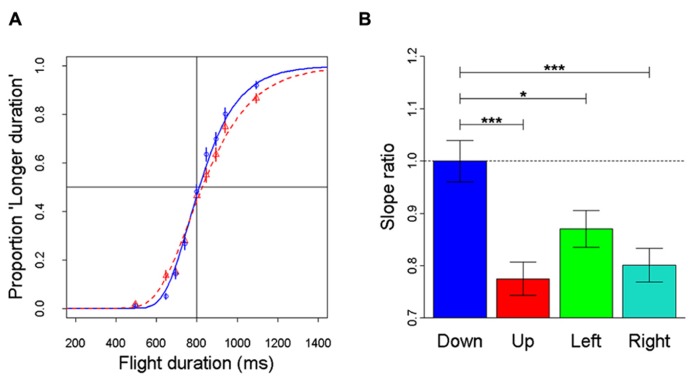
**Discrimination of visual motion duration.** In different trials, a target moved downward, upward, leftward, or rightward with constant acceleration (9.81 m s^-^^2^) and randomized initial speed, resulting in a variable total duration of motion. Observers judged whether the duration of the test stimulus was longer or shorter than a standard duration (800 ms). **(A)** Population psychometric functions for downward motion (blue) and upward motion (red). The graphs show the proportion of times the test stimulus appeared to last longer than the standard (data pooled over all participants). **(B)** For each motion direction, the precision of discrimination was assessed as the slope of the population response (all values normalized relative to the downward condition): the higher the slope, the greater the precision. Error bars: ± 1 SD. Significant differences: ***p < 0.001 and *p < 0.05. Replotted with permission from Moscatelli and Lacquaniti (2011).

**FIGURE 2:**
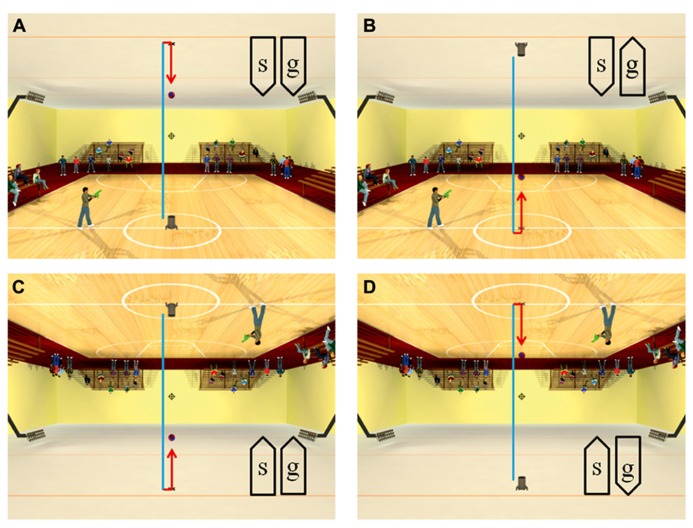
**Scenes displayed in the manipulation of visual congruence between background and gravity orientation.** The target ball was launched vertically from the launcher, hit the opposite surface and bounced back. The target decelerated from launch to bounce (blue trajectory), and it accelerated after bounce (red trajectory). Blue and red segments were not present in the actual movies. When the button was pressed, the standing character shot a bullet toward the interception point (indicated by the cross-hair). The direction of the scene (“s”) and the direction of gravity acting on the target (“g”) were varied in different blocks of trials: **(A)** normal scene and gravity, **(B)** normal scene and inverted target gravity, **(C)** inverted scene and gravity, **(D)** inverted scene and normal target gravity. Modified with permission from Zago et al. (2011b).

**FIGURE 3:**
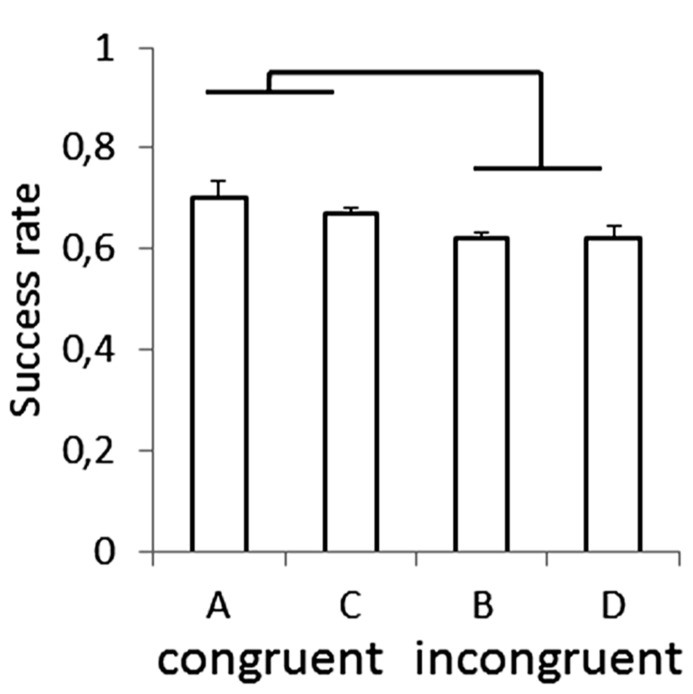
**Success rate for each type of scene in the manipulation of visual congruence between background and gravity orientation.** Brackets indicate that success rate was significantly (*p* < 0.05) higher for the congruent scenes (A,C in **Figure 2**) than for the incongruent ones (B,D). Modified with permission from Zago et al. (2011b).

Recently, anisotropies related to the direction of motion have been described in a task of time perception (Moscatelli and Lacquaniti, 2011). Observers were asked to judge the duration of motion of a target accelerating in one of four different directions, downward, upward, leftward, or rightward relative to a visual scene. Downward motion complied with the gravity constraint, whereas motion in the other directions violated this constraint. It was found that the precision of the duration estimates exhibited systematic anisotropies, the performance being significantly better for downward motion than for the other directions (**Figure 1**). The results demonstrated that prior knowledge about gravity force is incorporated in the neural mechanisms computing elapsed time. Similar mechanisms are at work when timing interception actions. Thus, Zago et al. (2011b) asked participants to press a button triggering a hitter to intercept a target accelerated by a virtual gravity. A factorial design assessed the effects of scene orientation (normal or inverted) and target gravity (normal or inverted, **Figure 2**). It was found that interception was significantly more successful when scene direction was concordant with target gravity direction, irrespective of whether both were upright or inverted (**Figure 3**).

## OBSERVATION OF BIOLOGICAL MOTION

Humans have evolved to recognize and interpret the behavior of other humans so as to interact with them effectively. Specialized mechanisms in the form of configural processing can help in the recognition process (Reed et al., 2012). For instance, changes in animate targets are detected faster than those in inanimate targets (New et al., 2007). Moreover, there is growing evidence that, to deal with animate motion, the brain uses mechanisms partially different from those used to deal with the motion of inanimate objects. The neural networks processing animate and inanimate targets are partially segregated in the brain (Caramazza and Shelton, 1998). This specialization takes advantage of the fact that the kinematics and dynamics of animals differs from those of passive objects on several counts (Zago et al., 2011a).

Recently, the hypothesis of specialized processing of animate and inanimate targets has been extended to encompass the temporal domain (Carrozzo et al., 2010). Namely, the hypothesis holds that there exist distinct time bases for animate and inanimate events. This specialization would enhance our ability to predict critically timed actions. When animacy is detected by a human observer, time is calibrated against the predictions regarding the motion of people and animals, allowing synchronization in inter-personal actions. When no animacy is detected, the time is calibrated against the predictions of motion of passive objects. This is consistent with the idea that time perception can be embodied, i.e., that affective and body states influence time judgments (Maniadakis and Trahanias, 2011).

**FIGURE 4:**
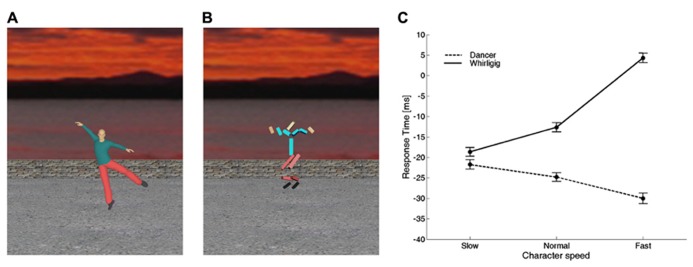
**Interference on timed responses by background motion of animate or inanimate figures. (A)** One frame from a movie of a dancer. Motion was captured from a real dancer performing several steps of classical ballet, and then rendered using computer graphics. **(B)** One frame from a movie of a whirligig. This consisted of disjointed rods, whose angular motion matched that of the corresponding body segment of the dancer. In different sessions, dancer and whirligig movements could be played at the normal recorded speed, at slow or fast speeds (corresponding to 0.5 and 1.5 times the normal one, respectively). **(C)** Average (±95% confidence intervals over all participants) response times for the slow, normal, and fast speeds. Modified with permission from Carrozzo and Lacquaniti (2013).

Consistent with this hypothesis, there is evidence that time perception and motor timing are influenced by animacy: the observation of a biological movement performed by other people biases the timing of a motor act or the judgment of perceived duration of an event (Watanabe, 2008; Bove et al., 2009; Carrozzo et al., 2010; Orgs and Haggard, 2011; Zago et al., 2011b,c; Mouta et al., 2012; Wang and Jiang, 2012; Carrozzo and Lacquaniti, 2013). In particular, Carrozzo et al. (2010) used interference paradigms in which a timing task was run concurrently with the presentation of different figures animated with computer-graphics in the background of the scene (**Figures 4A,B**). In separate experiments, they used two different timing tasks: (1) button-press responses aimed at intercepting a moving ball, and (2) discrimination of the duration of a stationary flash. The timing tasks served as probes to reveal biases or distortions of time induced by the background figures. In both tasks, the observers were presented with different background scenes before and during the execution of the task. The scene displayed figures which could differ in terms of biological (human) or non-biological appearance and kinematics. In all cases, the background figures and their movements were totally unrelated to the foreground target and to the viewer’s action. Carrozzo et al. (2010) found that, for both the motor interception and the time discrimination task, there was a systematic offset between the time estimates associated with biological movements and the time estimates associated with non-biological movements, consistent with the hypothesis that there exist timing mechanisms differentially tuned to these two sets of movements. In another study, the speed of the movements of the background figures was varied across sessions, so that the motion speed of all the segments of the character was scaled up or down by the same amount and to the same extent for both the biological and the non-biological figure (Carrozzo and Lacquaniti, 2013). The results confirmed the existence of an offset between the time estimates associated with biological movements and the time estimates associated with non-biological movements (**Figure 4**). Moreover, animation speed affected time estimates very differently for the two categories of movement: increasing the speed of the whirligig increased the delay of the responses considerably, whereas the effect of the dancer’s speed was weaker and in the opposite direction.

These results indicate that vision of human and inanimate motions exerts differential top-down influences on automatic processes computing time. Interference effects are observed when the background motion is unrelated to the task performed by the observer. By contrast, when the observed action is related and instrumental to the task performance, the interaction between the two (observed and performed) actions results in facilitation rather than interference (Sebanz and Knoblich, 2009). In this vein, Zago et al. (2011c) compared the timing of interception of a moving target when it depended on a biological motion or a non-biological motion triggered by the observer and simulated on the computer screen. They found that the timing significantly improved in the presence of biological movements under all ecological conditions of coherence between scene and target gravity directions. Also, visual discrimination of point-light motion of two interacting agents is worse when the two actions are desynchronized (Neri et al., 2006). In other words, time-locking in a behaviorally meaningful way between interacting agents provides an implicit temporal cue and the additional agent can be used to predict the expected trajectory of the relevant agent with better precision.

For biological motion, the correct timing of visual images is detected more accurately when motion flows in the normal forward direction. Thus, when muted video-clips of the lower face of speaking actors are shown at a variable rate, both faster and slower than the original rate, identification of the natural rate is accurate when the movies are played forward but not when they are played backward (Viviani et al., 2011a). Similarly, temporal reversals in dynamic displays of human locomotion are detected reliably only when they are played in the forward direction (Viviani et al., 2011b). Also these studies point to a specific tuning of time perception to biological movements. Implicit motor competence for the observed actions are presumably instrumental for extracting subtle discriminal information from the stimuli allowing correct temporal estimates.

In addition to real motion, also apparent motion and implied motion can affect time estimates (Orgs and Haggard, 2011). In particular, static images of an action convey dynamic information about previous and subsequent moments of the same action, and provide an impression of motion. Images with implied motion cause a forward displacement in spatial memory – a phenomenon known as representational momentum (Freyd, 1983). Implied motion also affects perceived time, as assessed with classical psychophysical methods. The duration of a visual stimulus conveying implied motion information is discriminated more precisely than a similar stimulus without implied motion (Moscatelli et al., 2011; Nather et al., 2011). Also, visual stimuli with implied motion produce time dilation just as real motion does (Nather et al., 2011; Yamamoto and Miura, 2012), although the distortion is smaller with implied motion. Indeed, when pictures depicting different sculptures of ballet dancers are shown, the duration is judged longer for the sculpture implying more movement than for the sculpture requiring less movement (Nather et al., 2011).

Expertise leads to a fine tuning of timing abilities. Professional pianists asked to reproduce the duration of visual displays outperform non-pianists when observing a specific action (a piano-playing hand), but not when observing non-specific actions (finger-thumb opposition; Chen et al., 2013). This indicates that musical expertise involves a selective dynamic internal representation that allows to estimate precisely the temporal duration of observed movements related to the expert performance. Similar results have been obtained by showing ballet steps to professional dancers: dancers were significantly less variable in their time estimations as compared to non-dancers (Sgouramani and Vatakis, 2013).

## MOTOR TIMING

According to one hypothesis, some of the processes involved in time perception, either a single internal clock, many specialized clocks, or a distributed network representations of time, are also used for timing motor commands (Treisman et al., 1992). As actions must often be coordinated with external events, it seems advantageous to use a shared representation for time perception and motor timing. Behavioral observations, such as correlations between interval discrimination thresholds and variability in the timing of repetitive tapping (Keele et al., 1985; Ivry and Hazeltine, 1995), similar interference patterns of sequences of auditory clicks at different frequencies on interval estimation and response time (Treisman et al., 1992), and significant transfer of training on a perceptual timing task to a motor timing task (Meegan et al., 2000), support the notion of shared timing mechanisms between perception and motor control. Also the study by Carrozzo et al. (2010) offers supporting evidence for shared perceptuo-motor timing. This study showed that the effects of an animate context were similar for the explicit perceptual judgment of duration and for the manual interception of a moving target, as were the effects of an inanimate context. These results suggested that, in both an automatic form of motor timing and a cognitive form of time perception, the observers became tuned to a time base intrinsically linked to a background character.

Imaging studies also suggest a shared neural substrate for perceptual and motor timing. For example, sustained perceptual analysis of auditorally and visually presented temporal patterns activates brain areas that are generally involved in motor preparation and coordination (Schubotz et al., 2000). However, not all timing tasks share the same timing mechanisms. Thus, timing variability is not correlated between repetitive tapping and continuous periodic drawing (Robertson et al., 1999; Zelaznik et al., 2002) and adaptation to visual motion may affect differently perception of interval duration and timing of anticipatory interceptive action (Marinovic and Arnold, 2012, but see Carrozzo et al., 2010). Moreover, the motor system uses a state representation instead of a time representation during adaptation to mechanical perturbations to arm movements (Conditt and Mussa-Ivaldi, 1999; Karniel and Mussa-Ivaldi, 2003).

**FIGURE 5:**
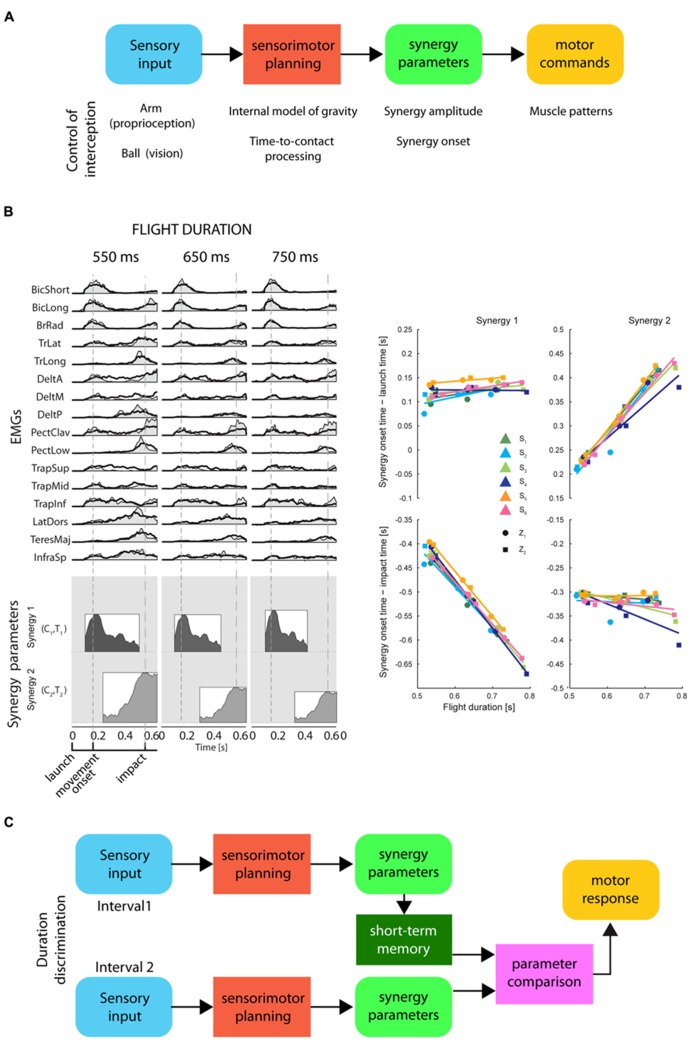
**Representation of timing for motor control and for perceptual discrimination by synergy parameters. (A)** Conceptual scheme of the information processing stages for the control of motor timing, i.e., movement duration and movement synchronization with external events, by a direct mapping of sensory input onto synergy recruitment parameters. These stages are illustrated in the example of the control of an interceptive movement: proprioceptive input about the arm posture and visual input about the ball trajectory are combined with *a priori* knowledge of gravity to predict the time-to-contact between ball and hand. The appropriate interceptive movement is then planned in terms of synergy recruitment parameters which are used to generate motor commands by modulating in amplitude and timing a set of muscle synergies. **(B)** Left: an example of muscle patterns (EMGs) recorded during catching of a ball flying with three different flight durations (columns) captured by modulation in amplitude and timing of two time-varying muscle synergies (coordinated recruitment of groups of muscles with specific activation profiles; the average profile of each synergy is illustrated as a shaded area within a rectangle on the bottom). Synergy amplitude and synergy onset time (synergy parameters) are illustrated by the height and the left edge of the rectangles, respectively [adapted from D’Andola et al. (2013)]. Notice that the onset of the first synergy is aligned with the ball launch and the onset of the second synergy with the impact of the ball with the hand. Right: a summary of the synergy onset timing for the two synergies (columns) with respect to launch time (top) and impact time (bottom) for six participants [adapted from D’Andola et al. (2013)]. **(C)** Conceptual scheme of a hypothetical interval duration discrimination process relying on short-term storage of synergy parameters for a planned movement synchronized with the events defining the intervals. Sensory input from the events defining the first interval are associated to synergy recruitment parameters for a motor plan synchronized with those events. These parameters are held in short-term memory until the parameters associated to the second interval are available for comparison.

On the timescale of a few hundreds of milliseconds, the perception of time elapsed between events may be related to movement planning and to the representation of movement duration. Simple movements of different durations often show kinematic regularities suggesting that duration is controlled adjusting a small number of parameters. For example, the spatial trajectory of point-to-point reaching movements is independent of movement duration and its tangential velocity is invariant when normalized for speed (Soechting and Lacquaniti, 1981; Atkeson and Hollerbach, 1985). Because of the non-linearity of the musculo-skeletal system, invariant kinematic features across movements with different durations require significant variation in the muscle patterns. However, the muscle patterns underlying movement with different spatial and temporal characteristics can be generated by scaling in amplitude and time and by shifting in time a small number of time-varying muscle synergies, i.e., coordinated recruitments of group of muscles with specific activation profiles (d’Avella and Lacquaniti, 2013). Invariant trajectories and speed profiles can be achieved by scaling the amplitude of time-normalized dynamic torque profiles by the square of the inverse of the movement duration (Hollerbach and Flash, 1982; Atkeson and Hollerbach, 1985), and similar scaling rules have been reported for time-varying muscle synergies (d’Avella et al., 2008). Thus, the control of movement duration may be achieved by setting the amplitude and the duration of a small number of time-varying muscle synergies (**Figure 5**).

When it is necessary to synchronize a movement with an external event, its duration must be selected according to a prediction of the future time occurrence of the event. Such prediction requires an internal model of the dynamic behavior of the physical entity or animate character associated with the event. An internal model may be implemented explicitly through a representation of the relevant variables and a simulation of their time evolution or, implicitly, as a mapping between sensory inputs and motor outputs generating the movement. In the latter case, a few spatio-temporal features of the sensory input may be directly mapped onto the amplitude and timing parameters modulating the recruitment of a few muscle synergies (see **Figure 5**; D’Andola et al., 2013). This strategy reduces the storage of the information relevant for temporal estimates to a low-dimensional mapping between sensory and motor signals. Therefore, elapsed time may also be represented by the synergy recruitment parameters for a movement which is synchronized to the sensory stimuli related to an external event. To judge the duration of an interval, the CNS might prepare a motor plan triggered by the stimulus signaling the onset of the interval, and synchronized with the stimulus which indicates the end of the interval. According to this hypothesis, when a discrimination between different time intervals is required, only the few synergy recruitment parameters encoding the duration of the motor plan associated to each interval would have to be compared.

## CONCLUSION AND PERSPECTIVES

The work reviewed here represents only a small fragment of a vast literature. Nevertheless, it suffices to indicate a very complex organization of both explicit time perception and implicit time estimates in humans. On the one hand, there is growing evidence for specialized mechanisms for time encoding in the sub-second range. One important specialization we considered is related to the animate–inanimate or living–non-living distinction. This distinction is a basic one, because it arises early in infancy, is cross-culturally uniform, and is critical for causal interpretations of events. Specialization of the neural time estimates presumably enhances the temporal resolution of sensory processing and the ability to estimate the duration of critical events. On the other hand, we emphasized the possibility that, although time perception is not unitary, there are some basic factors which can affect disparate time estimates in the same manner. Thus, we noticed that a neural time basis can be entrained by external cues in a similar manner for both perceptual judgments of elapsed time and in automatic motor control tasks. One possible reason underlying shared mechanisms for computing time is to be searched in the hypothesis that action observation involves an internal motor simulation of the observed movement (Prinz, 1997; Rizzolatti and Craighero, 2004; Choe et al., 2007). A motor resonance might derive from the synchronization of neural time to a base intrinsically linked to the internal simulation of the observed action. Thus, human perception of time may be strictly linked with the mechanisms used by humans to control their movements.

What is the relevance of all this for neurorobotics? Traditionally, the design and implementation of cognitive, sensory and motor abilities in robots depend on distinct fields of expertise. However, as we remarked at several points, the temporal dimension is shared by most sensory, motor, and cognitive tasks. One parsimonious solution, therefore, could be to implement in humanoids a unique architecture for dealing with time, which would apply the same specialized mechanisms to both perception and action, similarly to humans. There is the hope that this style of implementation might render the humanoids more acceptable to humans, thus facilitating reciprocal interactions.

An interesting idea that is emerging in parallel from biology and machine intelligence is that sensorimotor behaviors can be constructed from primitives, the most basic components of behavior (Poggio and Bizzi, 2004; Moro et al., 2012). For instance, we noticed above that several motor behaviors of humans appear to be built starting from elementary muscle synergies (d’Avella and Lacquaniti, 2013). There is also evidence that some such motor primitives are present at very early stages of human development, and they may be rooted in our evolutionary trajectory: indeed, these primitives appear to have been highly preserved and recombined during evolution (Dominici et al., 2011; Lacquaniti et al., 2013).

Choe et al. (2007) proposed that in robotics a developmental program can be based on a small number of non-ad hoc, biologically grounded principles which can spontaneously and autonomously give rise to models and goals within the artificial agent. According to this approach, the agent might develop and learn by starting to use rudimentary, initial, stereotypical motor primitives (akin to those found in human development) which would allow the agent to understand its own internal states in terms of its own actions, possibly by keeping internal state invariance (Choe et al., 2007). This approach would probably ensure that the time dimension is dealt with in a similar fashion in sensory-perceptual and motor processes, the premise with which we began this article.

## Conflict of Interest Statement

The authors declare that the research was conducted in the absence of any commercial or financial relationships that could be construed as a potential conflict of interest.

## References

[B1] Appelle S. (1972). Perception and discrimination as a function of stimulus orientation: the “oblique effect” in man and animals. *Psychol. Bull.* 78 266–278 10.1037/h0033117 4562947

[B2] Atkeson C. G. Hollerbach J. M. (1985). Kinematic features of unrestrained vertical arm movements. *J. Neurosci.* 5 2318–2330 4031998 10.1523/JNEUROSCI.05-09-02318.1985 PMC6565321

[B3] Ball K. Sekuler R. (1987). Direction-specific improvement in motion discrimination. *Vision Res.* 27 953–965 10.1016/0042-6989(87)90011-3 3660656

[B4] Bartneck C. Kuliæ D. Croft E. Zoghbi S. (2009). Measurement instruments for the anthropomorphism, animacy, likeability, perceived intelligence, and perceived safety of robots. *Int. J. Soc. Robot.* 1 71–81 10.1007/s12369-008-0001-3

[B5] Beckmann J. S. Young M. E. (2009). Stimulus dynamics and temporal discrimination: implications for pacemakers. *J. Exp. Psychol. Anim. Behav. Process.* 35 525–537 10.1037/a0015891 19839705

[B6] Bove M. Tacchino A. Pelosin E. Moisello C. Abbruzzese G. Ghilardi M. F. (2009). Spontaneous movement tempo is influenced by observation of rhythmical actions. *Brain Res. Bull.* 80 122–127 10.1016/j.brainresbull.2009.04.008 19394410

[B7] Breazeal C. (2003). Toward sociable robots. *Rob. Auton. Syst.* 42 167–175 10.1016/S0921-8890(02)00373-1

[B8] Brown S. W. (1995). Time, change, and motion: the effects of stimulus movement on temporal perception. *Percept. Psychophys.* 57 105–116 10.3758/BF03211853 7885802

[B9] Bruno A. Ayhan I. Johnston A. (2010). Retinotopic adaptation-based visual duration compression. *J. Vis.* 10 30 10.1167/10.10.30 20884495

[B10] Buhusi C. V. Meck W. H. (2005). What makes us tick? Functional and neural mechanisms of interval timing. *Nat. Rev. Neurosci.* 6 755–765 10.1038/nrn1764 16163383

[B11] Buffardi L. (1971). Factors affecting the filled-duration illusion in the auditory, tactual, and visual modalities. *Percept. Psychophys.* 10 292–294 10.3758/BF03212828

[B12] Burr D. Cicchini G. M. Arrighi R. Morrone M. C. (2011). Spatiotopic selectivity of adaptation-based compression of event duration. *J. Vis.* 11 21 10.1167/11.2.21 21357369

[B13] Calinon S. Guenter F. Billard A. (2007). On learning, representing, and generalizing a task in a humanoid robot. *IEEE Trans. Syst. Man Cybern. B Cybern.* 37 286–298 10.1109/TSMCB.2006.886952 17416157

[B14] Caramazza A. Shelton J. R. (1998). Domain-specific knowledge systems in the brain: the animate–inanimate distinction. *J. Cogn. Neurosci.* 10 1–34 10.1162/089892998563752 9526080

[B15] Carrozzo M. Lacquaniti F. (2013). Effects of speeding up or slowing down animate or inanimate motions on timing. *Exp. Brain Res.* 224 581–590 10.1007/s00221-012-3338-7 23161160

[B16] Carrozzo M. Moscatelli A. Lacquaniti F. (2010). Tempo rubato: animacy speeds up time in the brain. *PLoS ONE* 5:e15638 10.1371/journal.pone.0015638 PMC3012081 21206749

[B17] Chaminade T. Okka M. M. (2013). Comparing the effect of humanoid and human face for the spatial orientation of attention. *Front. Neurorobot.* 7:12 10.3389/fnbot.2013.00012 PMC3759784 24027525

[B18] Chaminade T. Hodgins J. Kawato M. (2007). Anthropomorphism influences perception of computer-animated characters’ actions. *Soc. Cogn. Affect. Neurosci.* 2 206–216 10.1093/scan/nsm017 18985142 PMC2569803

[B19] Chen Y. H. Pizzolato F. Cesari P. (2013). Observing expertise-related actions leads to perfect time flow estimations. *PLoS ONE* 8:e55294 10.1371/journal.pone.0055294 PMC3566219 23405131

[B20] Choe Y. Yang H. F Eng D. C. Y. (2007). Autonomous learning of the semantics of internal sensory states based on motor exploration. *Int. J. Hum. Rob.* 4 211–243 10.1142/S0219843607001102

[B21] Conditt M. A. Mussa-Ivaldi F. A. (1999). Central representation of time during motor learning. *Proc. Natl. Acad. Sci. U.S.A.* 96 11625–11630 10.1073/pnas.96.20.11625 10500227 PMC18084

[B22] Creelman C. D. (1962). Human discrimination of auditory duration. *J. Acoust. Soc. Am.* 34 528–593 10.1121/1.1918172

[B23] D’Andola M. Cesqui B. Portone A. Fernandez L. Lacquaniti F D’Avella A. (2013). Spatiotemporal characteristics of muscle patterns for ball catching. *Front. Comput. Neurosci.* 7:107 10.3389/fncom.2013.00107 PMC3735981 23966939

[B24] d’Avella A. Fernandez L. Portone A. Lacquaniti F. (2008). Modulation of phasic and tonic muscle synergies with reaching direction and speed. *J. Neurophysiol.* 100 1433–1454 10.1152/jn.01377.2007 18596190

[B25] d’Avella A. Lacquaniti F. (2013). Control of reaching movements by muscle synergy combinations. *Front. Comput. Neurosci.* 7:42 10.3389/fncom.2013.00042 PMC3630368 23626534

[B26] Dominici N. Ivanenko Y. P. Cappellini G. d’Avella A. Mondì V. Cicchese M. (2011). Locomotor primitives in newborn babies and their development. *Science* 334 997–999 10.1126/science.1210617 22096202

[B27] Eagleman D. M. (2008). Human time perception and its illusions. *Curr. Opin. Neurobiol.* 18 131–136 10.1016/j.conb.2008.06.002 18639634 PMC2866156

[B28] Eagleman D. Sejnowski T. J. (2000). Motion integration and postdiction in visual awareness. *Science* 287 2036–2038 10.1126/science.287.5460.2036 10720334

[B29] Eagleman D. M. Tse P. U. Buonomano D. Janssen P. Nobre A. C. Holcombe A. O. (2005). Time and the brain: how subjective time relates to neural time. *J. Neurosci.* 25 10369–10371 10.1523/JNEUROSCI.3487-05.2005 16280574 PMC6725822

[B30] Fraisse P. (1963). *The Psychology of Time*. New York: Harper and Row

[B31] Freyd J. J. (1983). The mental representation of movement when static stimuli are viewed. *Percept. Psychophys.* 33 575–581 10.3758/BF03202940 6622194

[B32] Gibbon J. (1977). Scalar expectancy theory and Weber’s law in animal timing. *Psychol. Rev.* 84 279–325 10.1037/0033-295X.84.3.279

[B33] Goodrich M. A. Schultz A. C. (2007). Human–robot interaction: a survey. *Found. Trends Human–Computer Interact.* 1 203–275 10.1561/1100000005

[B34] Haggard P. Clark S. Kalogeras J. (2002). Voluntary action and conscious awareness. *Nat. Neurosci.* 5 382–385 10.1038/nn827 11896397

[B35] Hammett S. T. Champion R. A. Morland A. B. Thompson P. G. (2005). A ratio model of perceived speed in the human visual system. *Proc. Biol. Sci.* 272 2351–2356 10.1098/rspb.2005.3239 16243695 PMC1559964

[B36] Hancock P. A. Weaver J. L. (2005). On time distortion under stress. *Theor. Issues Ergon. Sci.* 6 193–211 10.1080/14639220512331325747

[B37] Hansen B. C. Essock E. A. (2004). A horizontal bias in human visual processing of orientation and its correspondence to the structural components of natural scenes. *J. Vis.* 4 1044–1060 10.1167/4.12.5 15669910

[B38] Hietanen M. A. Crowder N. A. Ibbotson M. R. (2008). Differential changes in human perception of speed due to motion adaptation. *J. Vis.* 8 1–10 10.1167/8.11.6 18831600

[B39] Hollerbach M. J. Flash T. (1982). Dynamic interactions between limb segments during planar arm movement. *Biol. Cybern.* 44 67–77 10.1007/BF00353957 7093370

[B40] Ivry R. B. Hazeltine R. E. (1995). Perception and production of temporal intervals across a range of durations: evidence for a common timing mechanism. *J. Exp. Psychol. Hum. Percept. Perform.* 21 3–18 10.1037/0096-1523.21.1.3 7707031

[B41] Jarrassé N. Charalambous T. Burdet E. (2012). A framework to describe, analyze and generate interactive motor behaviors. *PLoS ONE* 7:e49945 10.1371/journal.pone.0049945 PMC3511490 23226231

[B42] Johnston A. Arnold D. H. Nishida S. (2006). Spatially localized distortions of event time. *Curr. Biol.* 16 472–479 10.1016/j.cub.2006.01.032 16527741

[B43] Kanai R. Paffen C. L. Hogendoorn H. Verstraten F. A. (2006). Time dilation in dynamic visual display. *J. Vis.* 6 1421–1430 10.1167/6.12.8 17209745

[B44] Kaneko S. Murakami I. (2009). Perceived duration of visual motion increases with speed. *J. Vis.* 9 1–12 10.1167/9.7.14 19761329

[B45] Karniel A. Mussa-Ivaldi F. A. (2003). Sequence, time, or state representation: how does the motor control system adapt to variable environments? *Biol. Cybern.* 89 10–21 12836029 10.1007/s00422-003-0397-7

[B46] Keele S. W. Pokorny R. A. Corcos D. M. Ivry R. (1985). Do perception and motor production share common timing mechanisms: a correctional analysis. *Acta Psychol. (Amst.)* 60 173–191 10.1016/0001-6918(85)90054-X 4091033

[B47] Lacquaniti F. Ivanenko Y. P. d’Avella A. Zelik K. E. Zago M. (2013). Evolutionary and developmental modules. *Front. Comput. Neurosci.* 7:61 10.3389/fncom.2013.00061 PMC3656358 23730285

[B48] Leiser D. Stern E. Meyer J. (1991). Mean velocity and total time estimation effects of order and proportions. *J. Environ. Psychol.* 11 347–358 10.1016/S0272-4944(05)80107-0

[B49] Lhamon W. T. Goldstone S. (1975). Movement and the judged duration of visual targets. *Bull. Psychon. Soc.* 5 53–54 10.3758/BF03336701

[B50] Macar F. (1996). Temporal judgments on intervals containing stimuli of varying quantity complexity and periodicity. *Acta Psychol. (Amst.)* 92 297–308 10.1016/0001-6918(95)00017-8

[B51] Maniadakis M. Trahanias P. (2011). Temporal cognition: a key ingredient of intelligent systems. *Front. Neurorobot.* 5:2 10.3389/fnbot.2011.00002 PMC3175600 21954384

[B52] Marinovic W. Arnold D. H. (2012). Separable temporal metrics for time perception and anticipatory actions. *Proc. Biol. Sci.* 279 854–859 10.1098/rspb.2011.1598 21900323 PMC3259939

[B53] Matthews W. J. (2011). How do changes in speed affect the perception of duration? *J. Exp. Psychol. Hum. Percept. Perform.* 37 1617–1627 10.1037/a0022193 21517218

[B54] Mauk M. D. Buonomano D. V. (2004). The neural basis of temporal processing. *Annu. Rev. Neurosci.* 27 307–340 10.1146/annurev.neuro.27.070203.144247 15217335

[B55] Meegan D. V. Aslin R. N. Jacobs R. A. (2000). Motor timing learned without motor training. *Nat. Neurosci.* 3 860–862 10.1038/78757 10966614

[B56] Minato T. Shimada M. Ishiguro H. Itakura S. (2004). “Development of an android robot for studying human–robot interaction,” in *Innovations in Applied Artificial Intelligence* eds Orchard R. Yang C. Ali M. (Berlin: Springer) 424–434

[B57] Mori M. (1970). The uncanny valley (translated from Japanese by Karl F. MacDorman and Takashi Minato). *Energy* 7 33–35

[B58] Moro F. L. Tsagarakis N. G. Caldwell D. G. (2012). On the kinematic motion primitives (kMPs): theory and application. *Front. Neurorobot.* 6:10 10.3389/fnbot.2012.00010 PMC3469981 23091459

[B59] Morrone M. C. Ross J. Burr D. (2005). Saccadic eye movements cause compression of time as well as space. *Nat. Neurosci.* 8 950–954 10.1038/nn1488 15965472

[B60] Mörtl A. Lawitzky M. Kucukyilmaz A. Sezgin M. Basdogan C. Hirche S. (2012). The role of roles: physical cooperation between humans and robots. *Int. J. Robot. Res.* 31 1656–1674 10.1177/0278364912455366

[B61] Moscatelli A. Lacquaniti F. (2011). The weight of time: gravitational force enhances discrimination of visual motion duration. *J. Vis.* 11 4 10.1167/11.4.5 21478379

[B62] Moscatelli A. Polito L. Lacquaniti F. (2011). Time perception of action photographs is more precise than that of still photographs. *Exp. Brain Res.* 210 25–32 10.1007/s00221-011-2598-y 21359660

[B63] Mouta S. Santos J. A. Lopez-Moliner J. (2012). The time to passage of biological and complex motion. *J. Vis.* 12 1–14 10.1167/12.2.21 22371437

[B64] Nather F. C. Bueno J. L. Bigand E. Droit-Volet S. (2011). Time changes with the embodiment of another’s body posture. *PLoS ONE* 6:e19818 10.1371/journal.pone.0019818 PMC3103514 21637759

[B65] Neri P. Luu J. Y. Levi D. M. (2006). Meaningful interactions can enhance visual discrimination of human agents. *Nat. Neurosci.* 9 1186–1192 10.1038/nn1759 16936721

[B66] New J. J. Scholl B. J. (2009). Subjective time dilation: spatially local, object-based, or a global visual experience? *J. Vis.* 9 1–11 10.1167/9.2.4 19271914

[B67] New J. Cosmides L. Tooby J. (2007). Category-specific attention for animals reflects ancestral priorities, not expertise. *Proc. Natl. Acad. Sci. U.S.A.* 104 16598–16603 10.1073/pnas.0703913104 17909181 PMC2034212

[B68] Nijhawan R. (1994). Motion extrapolation in catching. *Nature* 370 256–257 10.1038/370256b0 8035873

[B69] Nijhawan R. (2008). Visual prediction: psychophysics and neurophysiology of compensation for time delays. *Behav. Brain Sci.* 31 179–198 10.1017/S0140525X08003804 18479557

[B70] Nisky I. Avraham G. Karniel A. (2012). Three alternatives to measure the human-likeness of a handshake model in a Turing-like test. *Presence* 21 156–182

[B71] Orgs G. Haggard P. (2011). Temporal binding during apparent movement of the human body. *Vis. Cogn.* 19 833–845 10.1080/13506285.2011.598481

[B72] Pariyadath V. Eagleman D. (2007). The effect of predictability on subjective duration. *PLoS ONE* 2:e1264 10.1371/journal.pone.0001264 PMC2082074 18043760

[B73] Poggio T. Bizzi E. (2004). Generalization in vision and motor control. *Nature* 431 768–774 10.1038/nature03014 15483597

[B74] Prinz W. (1997). Perception and action planning. *Eur. J. Cogn. Psychol.* 9 129–154 10.1080/713752551

[B75] Reed C. L. Nyberg A. A. Grubb J. D. (2012). Contributions of visual and embodied expertise to body perception. *Perception* 41 436–446 10.1068/p7029 22896916

[B76] Rizzolatti G. Craighero L. (2004). The mirror-neuron system. *Annu. Rev. Neurosci.* 27 169–192 10.1146/annurev.neuro.27.070203.144230 15217330

[B77] Robertson S. D. Zelaznik H. N. Lantero D. A. Bojczyk K. G. Spencer R. M. Doffin J. G. (1999). Correlations for timing consistency among tapping and drawing tasks: evidence against a single timing process for motor control. *J. Exp. Psychol. Hum. Percept. Perform.* 25 1316–1330 10.1037/0096-1523.25.5.1316 10531665

[B78] Roelofs C. O. Z Zeeman W. P. C. (1951). Influence of different sequences of optical stimuli on the estimation of duration of a given interval of time. *Acta Psychol. (Amst.)* 8 89–128 10.1016/0001-6918(51)90007-8

[B79] Schaal S. (2007). The new robotics-towards human-centered machines. *HFSP J.* 1 115–126 10.2976/1.2748612 19404417 PMC2639844

[B80] Schiffman H. R. Bobko D. J. (1974). Effects of stimulus complexity on the perception of brief temporal intervals. *J. Exp. Psychol.* 103 156–159 10.1037/h0036794 4418727

[B81] Schmolesky M. T. Wang Y. Hanes D. P. Thompson K. G. Leutgeb S. Schall J. D. (1998). Signal timing across the macaque visual system. *J. Neurophysiol.* 79 3272–3278 9636126 10.1152/jn.1998.79.6.3272

[B82] Schubotz R. I. Friederici A. D Von Cramon D. Y. (2000). Time perception and motor timing: a common cortical and subcortical basis revealed by fMRI. *Neuroimage* 11 1–12 10.1006/nimg.1999.0514 10686112

[B83] Sebanz N. Knoblich G. (2009). Prediction in joint action: what, when, and where. *Top. Cogn. Sci.* 1 353–367 10.1111/j.1756-8765.2009.01024.x 25164938

[B84] Sgouramani H. Vatakis A. (2013). “Flash” dance: how speed modulates perceived duration in dancers and non-dancers. *Acta Psychol. (Amst.)* 1. 10.1016/j.actpsy.2013.06.009 23910150

[B85] Simoncelli E. P. Olshausen B. A. (2001). Natural image statistics and neural representation. *Annu. Rev. Neurosci.* 24 1193–1216 10.1146/annurev.neuro.24.1.1193 11520932

[B86] Smith A. T. Edgar G. K. (1994). Antagonistic comparison of temporal frequency filter outputs as a basis for speed perception. *Vision Res.* 34 253–265 10.1016/0042-6989(94)90337-9 8116284

[B87] Soechting J. F. Lacquaniti F. (1981). Invariant characteristics of a pointing movement in man. *J. Neurosci.* 1 710–720 7346580 10.1523/JNEUROSCI.01-07-00710.1981 PMC6564198

[B88] Sugimoto N. Morimoto J. Hyon S. H. Kawato M. (2012). The eMOSAIC model for humanoid robot control. *Neural Netw.* 29–30 8–19 10.1016/j.neunet.2012.01.002 22366503

[B89] Terao M. Watanabe J. Yagi A. Nishida S. (2008). Reduction of stimulus visibility compresses apparent time intervals. *Nat. Neurosci.* 11 541–542 10.1038/nn.2111 18408716

[B90] Thompson P. (1981). Velocity after-effects: the effects of adaptation to moving stimuli on the perception of subsequently seen moving stimuli. *Vision Res.* 21 337–345 10.1016/0042-6989(81)90161-9 7269311

[B91] Treisman M. (1963). Temporal discrimination and the indifference interval: implications for a model of the ‘internal clock’. *Psychol. Monogr.* 77 1–31 10.1037/h0093864 5877542

[B92] Treisman M. Faulkner A. Naish P. L. (1992). On the relation between time perception and the timing of motor action: evidence for a temporal oscillator controlling the timing of movement. *Q. J. Exp. Psychol. A* 45 235–263 10.1080/14640749208401326 1410557

[B93] Tse P. U. Intriligator J. Rivest J. Cavanagh P. (2004). Attention and the subjective expansion of time. *Percept. Psychophys.* 66 1171–1189 10.3758/BF03196844 15751474

[B94] Ulrich R. Nitschke J. Rammsayer T. (2006). Perceived duration of expected and unexpected stimuli. *Psychol. Res.* 70 77–87 10.1007/s00426-004-0195-4 15609031

[B95] Viviani P. Figliozzi F. Lacquaniti F. (2011a). The perception of visible speech: estimation of speech rate and detection of time reversals. *Exp. Brain Res.* 215 141–161 10.1007/s00221-011-2883-9 21986668

[B96] Viviani P. Figliozzi F. Campione G. C. Lacquaniti F. (2011b). Detecting temporal reversals in human locomotion. *Exp. Brain Res.* 214 93–103 10.1007/s00221-011-2809-6 21814834

[B97] Wang L. Jiang Y. (2012). Life motion signals lengthen perceived temporal duration. *Proc. Natl. Acad. Sci. U.S.A.* 109 673–677 10.1073/pnas.1115515109 PMC3306663 22215595

[B98] Watanabe K. (2008). Behavioral speed contagion: automatic modulation of movement timing by observation of body movements. *Cognition* 106 1514–1524 10.1016/j.cognition.2007.06.001 17612518

[B99] Weiss Y. Simoncelli E. P. Adelson E. H. (2002). Motion illusions as optimal percepts. *Nat. Neurosci.* 5 598–604 10.1038/nn0602-858 12021763

[B100] Xuan B. Zhang D. He S. Chen X. (2007). Larger stimuli are judged to last longer. *J. Vis.* 7 1–5 10.1167/7.10.2 17997671

[B101] Yamamoto K. Miura K. (2012). Time dilation caused by static images with implied motion. *Exp. Brain Res.* 223 311–319 10.1007/s00221-012-3259-5 22972451

[B102] Yarrow K. Haggard P. Heal R. Brown P. Rothwell J. C. (2001). Illusory perceptions of space and time preserve cross-saccadic perceptual continuity. *Nature* 414 302–305 10.1038/35104551 11713528

[B103] Zago M. Carrozzo M. Moscatelli A. Lacquaniti F. (2011a). Time, observation, movement. *Cogn. Crit.* 4 61–86

[B104] Zago M. La Scaleia B. Miller W. L. Lacquaniti F. (2011b). Coherence of structural visual cues and pictorial gravity paves the way for interceptive actions. *J. Vis.* 11 13 10.1167/11.10.13 21933933

[B105] Zago M. La Scaleia B. Miller W. L. Lacquaniti F. (2011c). Observing human movements helps decoding environmental forces. *Exp. Brain Res.* 215 53–63 10.1007/s00221-011-2871-0 21947172

[B106] Zago M. McIntyre J. Senot P. Lacquaniti F. (2009). Visuo-motor coordination and internal models for object interception. *Exp. Brain Res.* 192 571–604 10.1007/s00221-008-1691-3 19139857

[B107] Zakay D. (1992). “On prospective time estimation, temporal relevance, and temporal uncertainty,” in *Time, action, and cognition: towards bridging the gap* eds Macar F. Pouthas V. Friedman W. J. (Dordrecht: Kluwer Academic Press) 109–118

[B108] Zelaznik H. N. Spencer R. M. Ivry R. B. (2002). Dissociation of explicit and implicit timing in repetitive tapping and drawing movements. *J. Exp. Psychol. Hum. Percept. Perform.* 28 575–588 10.1037/0096-1523.28.3.575 12075889

